# Correction to: Dimensions of leisure-time physical activity and risk of depression in the “Seguimiento Universidad de Navarra” (SUN) prospective cohort

**DOI:** 10.1186/s12888-020-02549-5

**Published:** 2020-03-23

**Authors:** Alejandro Fernandez-Montero, Laura Moreno-Galarraga, Almudena Sánchez-Villegas, Francisca Lahortiga-Ramos, Miguel Ruiz-Canela, Miguel Ángel Martínez-González, Patricio Molero

**Affiliations:** 1 Department of Occupational Medicine, University of Navarra Clinic, Av. Pio XII, 36, 31008 Pamplona, Navarra Spain; 2 Department of Preventive Medicine and Public Health, University of Navarra, Pamplona, Spain; 3 IdiSNA (Instituto de Investigación Sanitaria de Navarra), Pamplona, Spain; 4 Department of Pediatrics, Complejo Hospitalario de Navarra, Servicio Navarro de Salud, Pamplona, Spain; 5 Nutrition Research Group, Research Institute of Biomedical and Health Sciences, University of Las Palmas de Gran Canaria, Las Palmas de Gran Canaria, Spain; 6 CIBER Fisiopatología de la Obesidad y Nutrición (CIBER Obn), Instituto de Salud Carlos III, Madrid, Spain; 7 Department of Psychiatry and Medical Psychology, University Clinic of Navarra, Pamplona, Spain; 8 Department of Nutrition, Harvard TH Chan School of Public Health, Boston, USA

**Correction to: BMC Psychiatry**

**https://doi.org/10.1186/s12888-020-02502-6**

After publication of our article [1] we have been notified that Table 2 was incorrectly formatted.

- Originally published version:

**Table 2:** Depression risk according to different dimensions of baseline physical activity. The SUN Project 1999–2018

**Total leisure-time physical activity** **(MET-h/wk)**				**< 10 MET-h/wk**
**Depression cases / person-years**	**366/59193**	**10 to 20 MET-h/wk**	**> 20 MET-h/wk**	**P for trend**
Multivariable-adjusted HR ^a^ (95% CI)	1 (ref.)	193/37606	311/66260	
**Time spent in leisure time physical activity (hours/wk)**	**< 75 min/wk**	0.88 (0.74–1.05)	0.84 (0.72–0.99)	0.046
Events / person-years	279/44420	**75–300 min/wk**	**> 300 min/wk**	
Multivariable-adjusted HR ^b^ (95% CI)	1 (ref.)	345/65624	246/53015	
**Intensity in leisure time physical activity (average METS)**	**Inactive**	0.88 (0.75–1.04)	0.83 (0.70–0.99)	0.057
Events / person-years	161/26004	**< 6 Average METs**	**≥6 Average METs**	
Multivariable-adjusted HR ^c^ (95% CI)	1 (ref.)	574/110834	135/26220	

^a^Adjusted for sex, baseline body mass index, time sleeping, time nap, time TV, total energy intake, adherence to the Mediterranean Diet, alcohol intake, smoking pack years, educational level, hypertension, diabetes mellitus, cancer and changes in physical activity in the 2th and 4th year follow-up, with age and year of entering the cohort as stratification variables

^b^Additional adjusted by intensity physical activity

^c^Additional adjusted by leisure time physical activity

- Correct version:

**Table 2:** Depression risk according to different dimensions of baseline physical activity. The SUN Project 1999–2018

**Total leisure-time physical activity (MET-h/wk)**	**< 10 MET-h/wk**	**10 to 20 MET-h/wk**	**> 20 MET-h/wk**	***P*for trend**
Depression cases / person-years	366/59193	193/37606	311/66260	
Multivariable-adjusted HR ^a^ (95% CI)	1 (ref.)	0.88 (0.74–1.05)	0.84 (0.72–0.99)	0.046
**Time spent in leisure time physical activity (hours/wk)**	**< 75 min/wk**	**75–300 min/wk**	**> 300 min/wk**	
Events / person-years	279/44420	345/65624	246/53015	
Multivariable-adjusted HR ^b^ (95% CI)	1 (ref.)	0.88 (0.75–1.04)	0.83 (0.70–0.99)	0.057
**Intensity in leisure time physical activity (average METS)**	**Inactive**	**< 6 Average METs**	**≥6 Average METs**	
Events / person-years	161/26004	574/110834	135/26220	
Multivariable-adjusted HR ^c^ (95% CI)	1 (ref.)	0.90 (0.75–1.09)	1.00 (0.79–1.27)	0.650

^a^Adjusted for sex, baseline body mass index, time sleeping, time nap, time TV, total energy intake, adherence to the Mediterranean Diet, alcohol intake, smoking pack years, educational level, hypertension, diabetes mellitus, cancer and changes in physical activity in the 2th and 4th year follow-up, with age and year of entering the cohort as stratification variables

^b^Additional adjusted by intensity physical activity

^c^Additional adjusted by leisure time physical activity

The original article has been corrected.

## References

[1] Fernandez-Montero A (2020). Dimensions of leisure-time physical activity and risk of depression in the “Seguimiento Universidad de Navarra” (SUN) prospective cohort. BMC Psychiatry.

